# The 4th NextGen therapies for SJIA and MAS: part 2 phenotypes of refractory SJIA and the landscape for clinical trials in refractory SJIA

**DOI:** 10.1186/s12969-023-00865-0

**Published:** 2024-01-05

**Authors:** Grant Schulert, Sebastiaan J. Vastert, Alexei A. Grom

**Affiliations:** 1 Division of Rheumatology, Cincinnati Children’s Hospital Medical Center, University of Cincinnati, Cincinnati, OH USA; 2 Department of Pediatric Rheumatology & Immunology and Center for Translational Immunology, University Medical Center Utrecht, Utrecht, the Netherlands

**Keywords:** IL-1, IL-6, IL-18, Refractory SJIA, Refractory SJIA Trial Design, Persistent partial MAS, SJIA-LD

## Abstract

Although the introduction of the IL-1 and IL-6 inhibiting biologics in 2012 has revolutionized the treatment and markedly improved outcomes for many patients with SJIA, about 20% of these patients continue to have active disease, have markedly decreased quality of life and high disease activity as well as treatment-related morbidity and mortality. There is a clear need to define these disease states, and then use these definitions as the basis for further studies into the prevalence, clinical features, and pathophysiologic mechanisms. While such patients are most likely to benefit from novel therapies, they are very difficult to enroll in the ongoing clinical trials given the unique features of their disease and large numbers of background medications. The discussions at the NextGen 2022 conference focused on strategies to overcome these obstacles and accelerate studies in refractory SJIA.

## Introduction

Although systemic JIA has historically been considered the most severe category of JIA, the introduction of the IL-1 and IL-6 inhibiting biologics in 2012 has revolutionized the treatment and markedly improved outcomes for many patients. Both Childhood Arthritis and Rheumatology Research Alliance (CARRA) and the Pediatric Rheumatology European Society (PReS) have undertaken longitudinal cohort registry studies to understand how biologic therapies for SJIA perform in real life [1–3]. Through these initiatives, as well as other long term follow-up studies like the Nordic cohort and Canadian ReACCh-Out cohort, it has become clear that even in 2022, a substantial subset of children with SJIA can be treatment-refractory, necessitating sequential trial of multiple medications with chronic corticosteroid dependence [4–6]. The substantial morbidity and mortality among these patients underlined the critical need for new treatment approaches prompting clinicians, researchers, patients and parents to focus on this group of patients. However, progress in this area has been limited by an incomplete understanding of the nature refractory SJIA, a lack of broadly accepted and validated definitions of refractory SJIA as well as difficulty of enrolling these patients in clinical trials with traditional designs.

## Defining refractory SJIA

An international consensus on the definition of refractory SJIA does not exist but has been an important topic at conferences and meetings. Such definitions are urgently needed to define inclusion criteria for international cohort studies of these patients and eventually clinical trials to better understand pathogenesis and improve the treatment. The *Systemic JIA Foundation* (www.systemicjia.org) has been instrumental in boosting such collaborations, not only by organizing meetings (such as this) focused on clinical and research initiatives aiming to gain insight in refractory SJIA, but also by setting up patient-driven research initiatives [7]. In clinical practice, there are at least four distinct clinical patterns of refractory disease that are considered under the umbrella term of “refractory SJIA”. Although these phenotypes may clinically overlap, they still appear to differ in the underlying biology of disease, morbidity, mortality, and potential therapeutic approaches. These phenotypes include (1) refractory SJIA with predominantly persistent arthritis; (2) refractory SJIA with predominantly systemic features; (3) refractory SJIA with chronic parenchymal lung disease (SJIA-LD); and (4) refractory SJIA with relapsing MAS (including patients with predominantly liver involvement). Several preliminary definitions have recently been proposed by expert groups and with the input of patients and families. Some of these definitions are summarized in Table 1.

**Table 1:** Definitions of refractory SJIA

Clinical phenotype	Proposed definition	Reference
Refractory SJIA (broadly)	Failure to respond to IL-1 AND IL-6 blocking biologics (failure = inability to resolve arthritis, systemic symptoms, or liver dysfunction, or being glucocorticoid dependent) OR ≥ 2 episodes of MAS in a 2 year period OR Development of SJIA-LD	Canna et al. (2020) [7]
Persistent partial MAS	A patient with active SJIA and persistent inflammation (provided that infection or other causes have been ruled out) and newly worsening or persistently abnormal values for at least 6 weeks (with inability to taper medications because of worsening values) indicating: - Liver abnormalities - Disorder of hematopoiesis - Coagulopathy - Highly elevated serum IL-18 with modestly elevated CXCL9	Canna et al. (2020) [7]
Refractory SJIA arthritis	SJIA patients whose arthritis fails to respond to both IL-1 and IL-6 therapy, defined as continued arthritis disease activity requiring maintenance therapy with glucocorticoids	Erkens et al. (2021) [8]
Refractory/recurrent SJIA-associated MAS	SJIA related MAS, requiring long term adjunctive therapy with glucocorticoids, OR *Recurrent* (≥ 2 episodes of) SJIA related MAS	Erkens et al. (2021) [8]
SJIA-LD	*Suspected SJIA-LD*: Objective findings on clinical exam (including but not limited to tachypnea, cough, or clubbing); OR diffuse abnormalities on chest imaging* *Probable SJIA-LD*: both clinical findings and chest imaging findings as above, OR pulmonary hypertension as measured by echocardiogram. *Definite SJIA-LD*: tissue biopsy consistent with ILD, PAP/ELP, or PAH. *not due to lung disease that preexisted SJIA diagnosis, infection, or other identifiable cause.	Erkens et al. (2021) [8]

## Summary of presentations

Dr. Alexei Grom started with the description of the distinct clinical patterns of refractory SJIA. By his estimation, at least 20% of SJIA patients do not respond to the existing treatments well and develop a refractory disease course. These patients continue to have persistent disease activity requiring maintenance therapy with high dose glucocorticoids. The patients with refractory SJIA not only have high mortality but also have disease- and medication-related morbidities that slowly accumulate affecting all aspects of the child’s lifetime quality of life. As the degree of immunosuppression needed in most of these patients is not sustainable in the long term, all refractory SJIA families are anxious to gain access to new biomarkers for early diagnosis and new treatments. Notably and unfortunately, many of these patients, particularly those without arthritis who do not meet the ILAR diagnostic criteria for SJIA, are not eligible for the ongoing in clinical trials in SJIA.

### Refractory SJIA with arthritis

Although patients with “*refractory SJIA with arthritis*” usually do meet the diagnostic criteria for SJIA, they are typically treated with multiple biologic and non-biologic DMARDs and it is not clear how to handle these background medications at enrollment given frequent requirements for medication wash-out periods and prohibition of concomitant biologic treatment.

### The second category of refractory SJIA are patients without arthritis

Who tend to have prominent and persistent systemic features. In many of them the rash is often atypical, highly pruritic and reminiscent of urticaria. In Dr. Grom’s experience, these patients tend to have higher rates of MAS, and strikingly high levels of serum IL-18 (often exceeding 50,000 pg/ml). In general, these patients are very difficult to enroll in the ongoing clinical trials for several reasons. First, patients who have never had arthritis cannot be diagnosed with definitive SJIA under ILAR or CARRA provisional definitions [9, 10]. The other challenge in this group of patients is that the existing outcome measures such as the ACR JIA response criteria [11] are focused predominantly on the arthritis and do not capture well the systemic component of the disease. Potential solutions may include modifying the existing ACR JIA response measures; improving systemic JADAS, or applying the auto inflammatory disease activity index [12].

### The third clinical pattern in refractory SJIA is SJIA-LD

These patients tend to present with first signs of SJIA before the second birthday, and they typically have more prominent and persistent systemic features and less arthritis [13, 14]. About 80% of these patients have a history of MAS, often recurrent, and about 40% develop reactions to biologic medications [14]. SJIA-LD patients also tend to have very high levels of IL-18 that rise further during disease flares. When these patients are diagnosed with the lung disease, there is a striking dissociation between relatively mild clinical findings and the advanced changes on CT scans, including pleural thickening, consolidation with bronchial vascular centric tree-in-bud opacities, intralobular septal thickening, ground-glass opacities and eventually areas of consolidation [13, 14]. Histopathologically SJIA-LD is very distinctive with patchy but often extensive lesions comprised of mixed features of pulmonary alveolar proteinosis and endogenous lipioid pneumonia as well as massive interstitial infiltration by lymphoplasmacytic cells including T cells [13, 14]. Other characteristic features include pleural and intralobular septal collagenous fibrosis and occasionally vasculopathy that may lead to pulmonary hypertension. These patients are very difficult to enroll into clinical trials for several reasons: first, the absence of arthritis may preclude the diagnosis of definitive SJIA; second, these patients have numerous background medications that may be difficult to handle at enrollment; and third, the outcome measures for the lung component of the disease have not been well defined.

### The fourth clinical pattern of refractory SJIA includes patients with relapsing MAS

Approximately 5% of patients with MAS may have frequent or almost continuous episodes of MAS; this pattern has been also described as “persistent partial MAS” [7]. Again, these patients tend to have very high levels of serum IL-18 and are at a high risk for SJIA-LD. This group also includes very perplexing patients who have predominantly liver involvement. These patients typically have markedly, and persistently elevated liver enzymes associated with laboratory features of overt or subclinical MAS. The histopathologic findings on liver biopsy in this group include sinusoidal inflammatory infiltrates that consist of T lymphocytes (predominantly CD8+) as well as CD163 + macrophages. These CD163 + macrophages represent highly activated Kupffer cells secreting proinflammatory cytokines and exhibiting hemophagocytic activity. A recent report from Dr. de Benedetti’s group [15] comprehensively described three patients with this histopathological pattern. Interestingly, all three patients exhibited gene expression signatures indicating T cell activation and massive production of IFN-γ. As these patients are relatively rare, it would be difficult to design a trial focused on this specific phenotype only. One possible solution is to include them as a subgroup in a larger trial with a “basket design”.

## Summary of discussions

There followed a discussion moderated by Dr. Hermine Brunner that included FDA representatives regarding how to include refractory SJIA patients in clinical trials, particularly patients who lack arthritis as a prominent disease manifestation. A “basket trial” with open label treatment that would enroll patients with different phenotypes including GI, lung, or liver diseases was considered. The main challenge with this approach, however, would be the assessment of efficacy. Dr. Brunner suggested demonstrating efficacy with older patients in a placebo-controlled setting as a first step followed by open-label treatment for younger patients. Dr. Benedetti suggested the “*N* = 1 internal control design” as an alternative. The group, however, agreed that the best option would be an open label study with an external historical control cohort with matching inclusion criteria. The existing published cohorts, however, would not satisfy the FDA requirements, and a more comprehensive natural history study is necessary.

The second part of the discussion was focused on potential outcome measures. Dr. Grom suggested the option of assessing the ability to taper and/or discontinue medications such as glucocorticoids, Cyclosporin A, or etoposide. From the FDA perspective, however, reductions in other medications could be considered a secondary endpoint, but it would still be necessary to measure the direct clinical benefit of the experimental treatment. FDA does consider reduction of toxic medications, including steroids, as supporting evidence for efficacy, but it still needs primary endpoints based on clinical outcomes. In other words, outcomes such as reducing or even discontinuing steroids are driven by the direct clinical benefits of the experimental treatment that needs to be captured– whether it is clinical features or levels of inflammatory markers including IL-18. Dr. Sinha noted that reduction in medication usage per se may increase patient quality of life, and patients who were on less immunosuppression would be able to go to school and have more social interactions. Therefore, there was an agreement that patient-reported outcomes also needed to be included in the assessment of the efficacy of an experimental treatment, perhaps as secondary endpoints.

The discussion then turned to the challenges related to development of inclusion criteria for trials in rare diseases. Successful trials in monogenic diseases with a known genetic defect such as NLRC4-GOF or DIRA were used as examples. The question was whether in a rare disease like SJIA-LD without a known genetic cause, similar approaches could be used. From the FDA perspective in DIRA trials, the treatment with IL-1 blockade was essentially used as a replacement therapy in a disease with a well characterized genetic mechanism. Therefore, it might not be comparable to a more complex condition like SJIA. Dr. Sinha posed a question about how the FDA approached the assessment of the risk-benefit ratio for an experimental treatment in a severe life-threatening disease where the risk of “no treatment” is so high. From the FDA perspective, in this setting, although there might be less focus on the risk, the critical need to demonstrate the benefit of the drug remains, as it was important to ensure that a medication was not giving a false promise. Dr. De Benedetti noted that these measurements could have multiple forms, including physician global assessments and markers of inflammation such as CRP and ferritin. However, there is still a need for a primary outcome capturing clinical features that are indeed relevant to the disease studied.

## Summary and future directions

Refractory disease courses of SJIA are a significant clinical problem as these patients have markedly decreased quality of life and very high disease and treatment-related morbidity and mortality. From a research standpoint the first step is to define these disease states, and then use these definitions as the basis for further studies into the prevalence, clinical features, and pathogenesis. While such patients are most likely to benefit from novel therapies, they are very difficult to enroll in the ongoing clinical trials given the unique features of their disease and large numbers of background medications. As such, patient-driven efforts to accelerate studies in refractory SJIA should be a key priority of the research community.

## Data Availability

All the data discussed during the meeting have now been published and appropriately referenced at the end of the manuscript.

## Abbreviations

ACR: American College of Rheumatology
CARRA: Childhood Arthritis and Rheumatology Research Alliance
CRP: C-Reactive Protein
CT: Computerized tomography
DIRA: Deficiency of interleukin-1 receptor antagonist
DMARDS: Disease-modifying anti-rheumatic drug
GI: Gastrointestinal
IFN-γ: Interferon Gamma
IL: Interleukin
ILAR: International League of Associations for Rheumatology
JIA: Juvenile Idiopathic Arthritis
LD: Lung disease
MAS: Macrophage activation syndrome
PReS: Pediatric Rheumatology European Society
SJIA: Systemic Juvenile Idiopathic Arthritis
FDA: U.S. Food and Drug Administration
